# Supplementary material for: A Self-Healing Ionic Liquid-Based Ionically Cross-Linked Gel Polymer Electrolyte for Electrochromic Devices
Source: Polymers (Basel). 2021 Feb 27;13(5):742. doi: 10.3390/polym13050742 (PMC7957721; doi:10.3390/polym13050742)
Supplement: Supplementary file 1 [file polymers-13-00742-s001.pdf]

Supplementary material

A self-healing ionic liquid-based ionically crosslinked gel polymer electrolyte for electrochromic devices

Wanyu Chen [1], Siyuan Liu [1], Le Guo [2], Guixia Zhang [1], Heng Zhang [1], Meng Cao [1], Lili Wu [1], Tianxing Xiang [2] , Yong Peng [2,*]

In all the samples, acrylic acid and 2-diethylaminoethyl methacrylate were added as ion associate. Ionically crosslinked gel networks were formed by copolymerization of ion associate and different monomers. The synthesis were completed via following step: 1. The monomers and ion associate (AA, DEA) are mixed in a volume ratio of 8:1:1 to form precursors. 2. Ionic liquids and precursors were mixed uniformly with a volume ratio of 2:1. 3. A certain amount of initiator and LiTFSI was added to the ILs and precursors mixture. 4. This mixture were ultrasonicated for 1.5h, then the solution was bubbled with nitrogen for 30 minutes to ensure an O_2-free environment, and then exhausted the remaining gas dissolved in the system. 6. This O_2 free solution was transferred to a sealed mold composed of two pieces of FTO glass and this mold was then heat treated at an appropriate temperature (The heat treatment time varies according to different monomers and initiators).

Table S1 Transparency of different components of ionically crosslinked GPEs

	[Bmim][BF_4]	[Bmim][$EtSO_4$]	[Bmim][TFSI]
MMA	O	O	T
ACN	O	O	O
VAc	U	U	U
St	O	O	O
HEMA	O	U	O
AAm	O	O	U

MMA: Methyl methacrylate, ACN: Acrylonitrile, VAc: Ethenyl ethanoate, St: Styrene,

HEMA: 2-Hydroxyethyl methacrylate, AAm: Acrylamide.

O: Opaque, T:Transparent, U:Unable to polymerization.

As shown in Figure S1, the cycling durability of the ECD was studied by applying a periodically positive and negative voltage of 3.0 V for 200 cycles. The transmittance difference of ECD under bleached and colored states is 49.9% before applying repetitive voltage bias. After 100 cycles, the difference reduces to 42.1%, which reduces only 7.8% of its initial value. After 200 cycles, the difference reduces to 35%.

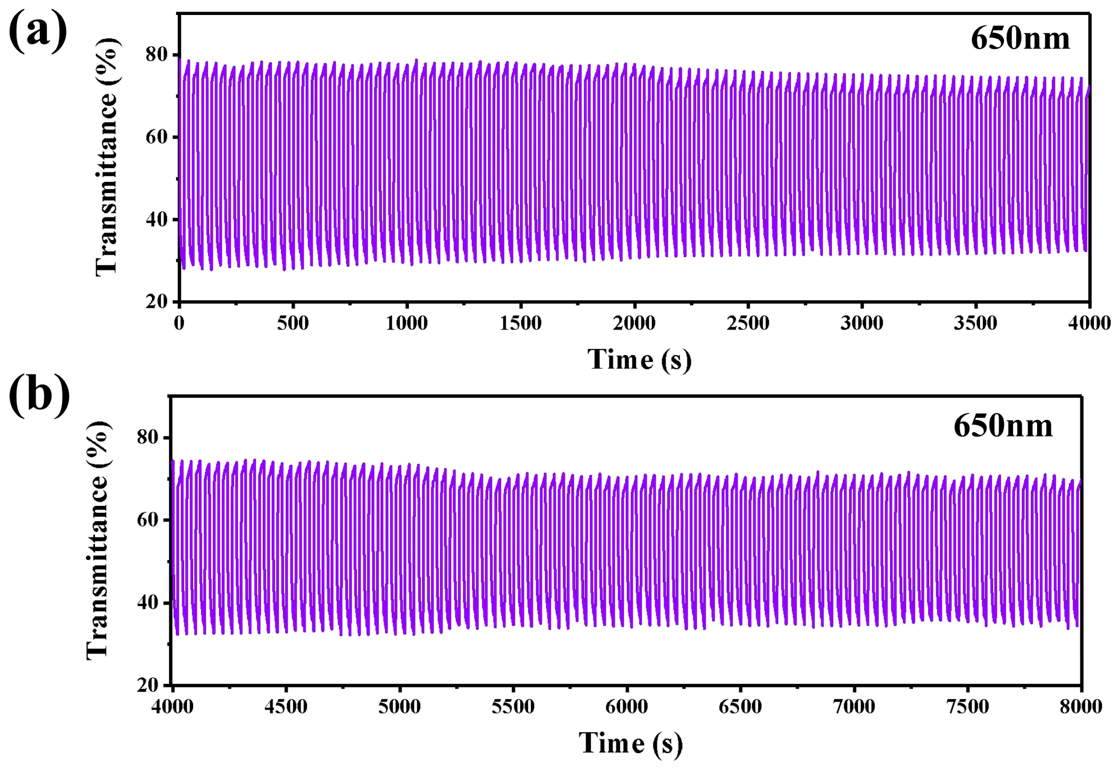

Figure S2 In situ optical response of ECD measured at 650 nm. (a) 1-100th cycles (b) 100-200th cycles. Potential: 3.0 V, cycling time: 40 s.

The video of ECD in coloring and bleaching processes and the stretching of GPE-ILs is provided.
